# Polymorphisms in the PVT1 Gene and Susceptibility to the Lung Cancer in a Chinese Northeast Population: a Case-control Study

**DOI:** 10.7150/jca.34320

**Published:** 2020-01-01

**Authors:** Ziwei Zhang, Hang Li, Juan Li, Xiaoting Lv, Zitai Yang, Min Gao, Yanhong Bi, Shengli Wang, Zhigang Cui, Baosen Zhou, Zhihua Yin

**Affiliations:** 1 Department of Epidemiology, School of Public Health, China Medical University, Shenyang 110122, PR China;; 2 Key Laboratory of Cancer Etiology and Intervention, University of Liaoning Province, Shenyang 110122, PR China;; 3 School of Nursing, China Medical University, Shenyang 110122, China.

**Keywords:** lung cancer, lncRNA, PVT1, single nucleotide polymorphism, susceptibility

## Abstract

**Background**: Long non-coding RNA (lncRNA) PVT1 has been identified to be related to risk of a variety of cancers, such as gastric cancer, pancreatic cancer and follicular lymphoma. This study assesses the association between genetic polymorphisms of PVT1 and the susceptibility to lung cancer as well as gene-environmental interaction.

**Method:** A hospital-based case-control study, including 515 lung cancer patients and 582 healthy controls, was carried out in Shenyang, China. Unconditional logistic regression analyses calculated the odds ratios (ORs) and their 95% confidence intervals (CIs) to assess the associations between polymorphisms of rs2608053, rs1561927, rs13254990 and susceptibility to lung cancer. The gene-environment interaction was evaluated by additive model and multiplicative model.

**Results:** There were no statistically significant associations between rs2608053 and rs1561927 polymorphisms in PVT1 and risk of lung cancer in the overall population. The relationship between polymorphism rs13254990 in PVT1 gene and lung adenocarcinoma was significant. Composed with individuals carrying CC genotypes, TT genotype carriers were more likely to develop lung adenocarcinoma (adjusted OR=2.095; 95%CI=1.084-4.047, P=0.028). In the recessive model, it also showed a statistically significant difference (TT vs CT+CC: adjusted OR=2.251, 95%CI=1.174-4.318, P=0.015). In nonsmokers, individuals carrying genotype CT had a lower risk of lung cancer than those with CC genotype (adjusted OR=0.673, 95%CI=0.472-0.959, P=0.028). Comparing with the homozygous CC, the patients with the heterozygous CT had a lower risk of NCSLC in the non-smoking group (adjusted OR =0.685, 95%CI=0.477-0.984, P=0.040). Additionally, gene-environment interaction results were not statistically significant in either additive model or multiplicative model.

**Conclusion**: The polymorphism rs13254990 in PVT1 gene is associated with the risk of lung adenocarcinoma in a Chinese northeast population.

## Introduction

As a heterogeneous, complex and intractable malignant tumor, lung cancer has brought enormous families' economic burden and jeopardized social health, which has gradually become one of the most important public health problems worldwide. According to the GLOBOCAN 2018 estimates of cancer incidence and mortality by the International Agency for Research on Cancer, lung cancer is the leading cause of cancer deaths both in China and the world^1^. Data indicated that an estimated 2.1 million new cases of lung cancer and 1.8 million deaths in global, while in China the number was 0.7 million and 0.6 million respectively^2^. Due to the rapidly increasing cases, it is urgent to figure out effective scientific methods to prevent the risk of lung cancer, improving patients' treatment and prolonging the survival time of patients. The tumorigenesis and development of lung cancer is related to behavioral, environmental and genetic discrepancies, among which cigarette smoking is the primary risk factor^3^. Nevertheless,10%-25% of lung cancer patients are nonsmokers in the world, which shows that the individual genetic factor also plays an important role in carcinogenesis of lung cancer^4^.

With the emergence of molecular epidemiology, more and more attention is focusing on common RNA species: non-coding RNAs (ncRNA), as most of the human genome nucleotides can be transcribed for primary transcripts of different cells to produce a range of them by ENCODE pilot project^5^. LncRNAs are one of general non-coding RNAs with a length >200 nt, lacking apparent open-reading frames (ORFs)^6^. On the basis of the GENCODE v7 analysis, lncRNAs have the characteristics of weaker evolutionary constraint and lower expressed, compared with coding genes. It also had canonical gene structures similar to mRNA^7^. Moreover, lncRNAs are preferentially enriched in the nucleus of the cells after transcription, which are involved in nuclear epigenetic modifications and transcriptional regulation of repetitive DNA elements^8^. Because of these multiple features, lncRNAs are abundantly expressed and widely related to a diversity of cancers. Especially, the aberrant expression and mutations of lncRNAs are associated with tumorigenesis, metastasis and tumor stage 9. Hence, we are able to predict that lncRNAs may be linked to carcinogenesis and promotion of lung cancer.

PVT1 was initially discovered in the mid-80s as a breakpoint site in murine plasmacytomas variant translocations^10^. Its locus was also identified as a retroviral integration site for leukemia virus (MLV)-induced T lymphomas of mice and rats and the analogous of Burkitt's lymphomas in human. PVT1, 1716 nt in length, is located in human chromosomal location 8q24.21 and starts about 57kb downstream of well-known MYC oncogene^11^. PVT1 is involved in both physiological and pathological processes, in particular, the tumorigenesis in multifarious cancer, presenting the aberrant level of PVT1 in proliferation, angiogenesis and metastasis of human malignancy^12^, ^13^. Although the detailed mechanism of PVT1 remains incomplete, decades of study have identified that some molecular functions may influence carcinogenesis by three main pathways. First, intron and exon of PVT1 exhibit the ability to promote tumorigenesis because it participates in interfering with the regulation of multiple oncogenes and anti-oncogenes by DNA rearrangements^14^, ^15^. Second, the PVT1 locus encodes a range of non-coding RNAs as well as generates a cluster of six annotated microRNAs (namely, miR-1204, miR-1205, miR-126, miR-1207-5p, miR-1207-3p and miR-1208) which can aberrant express in cancer or act as oncogenes or tumor suppressors^16^. Third, PVT1, an activator of oncogene MYC transcription^16^, regulates MYC in several different kinds of way. Another study showed that PVT1 affects the protein stability of MYC by Annexin-A2(ANXA2), a kind of Ca2+-binding protein, which binds directly to both ribonucleotide homopolymers and MYC RNA to promote cell proliferation in cancer^17^, ^18^. Simultaneously, several studies had shown that PVT1 is upregulated in various human tumors including gastric cancer 19, hepatic carcinoma 20, and prostate cancer^21^. Therefore, we pay our attention to investigating whether PVT1 polymorphisms may be involved in the risk of lung cancer.

Using high-throughput technologies, genome-wide association studies (GWAS) have shown evidence of more than one-third of single nucleotide polymorphisms (SNPs) lying within the lncRNAs^22^, which may affect gene expression and function and consequently augment the risk of cancer^23^, ^24^. Accumulating study demonstrated that PVT1 polymorphisms were associated with the risk of various kinds of malignant tumors such as Hodgkin lymphoma^25^, pancreatic cancer^26^, and follicular lymphoma^27^. Moreover, the studies on the association between the SNPs in lncRNAs and the susceptibility of lung cancer were few so far. Thus we perform a hospital-based case-control study consisting of 515 lung cancer cases and 582 cancer-free controls to evaluate the relevance of the three SNPs and susceptibility of lung cancer.

## Materials and Methods

### Study subjects and data collection

Our research, a hospital-based case-control study, was carried out in Shenyang city which is situated in Liaoning province at the northeast of China. Based on the strict inclusion and exclusion criteria, 515 patients diagnosed with lung cancer (from January 2014 to January 2017) were included in the study. The criteria were as follows: (a) patients were newly diagnosed with lung cancer or examined through histopathological examination; (b) patients with no previous cancer or metastatic cancer; (c) patients with no radiotherapy and chemotherapy before diagnosed. Likewise, 582 subjects were enrolled in control group from medical examination centers of the same hospital, matched to case group on age (±5 years).Subjects devoted 5 ml of venous blood and adopted unified questionnaire to gain the baseline characteristics of all individuals, including gender, age, smoking exposure and so on. Each participant was unrelated ethnic Han Chinese and had signed the informed consent form. Additionally, subjects who have smoked more than 100 cigarettes in their lifetime are identified as smokers while others are defined as non-smokers. The study was approved by the Institutional Review Board of the China Medical University.

### SNP selection and genotyping

By gathering the data of Han Chinese from the 1000 Genome Projects, we gain the genomic information about PVT1 gene, such as genomic sequence, genomic regions, etc. We utilized the Haploview 4.2 software with unified standard to detect the tagSNPs of PVT1. Combining the previous studies of PVT1 polymorphisms on the risk of cancer, we selected three SNPs (rs2608053, rs1561927, rs13254990) to conduct the study ultimately. TaqMan probe, primers and Master Mix were designed and manufactured by Invitrogen (Applied Biosystems). The Genomic DNA samples were extracted from the peripheral blood of the subjects by Phenolchloroform Method. Before utilization, all reagents were stored at -20°C. Furthermore, we selected an Applied Biosystems 7500 FAST Real-Time PCR System (Foster City, CA, USA) to conduct PCR reaction and gain fluorescent signal reading. Genotyping results of the three SNPs were presented on the Allelic Discrimination system of SDS software by labeling FAM and VIC fluorescence intensity. For strict quality control, we randomly selected 10% of the samples to check information integrity and accuracy and conducted duplicate experiments. All the information and results were in accordance.

### Statistical analysis

Considering that the age variable was a continuous variable and gender and smoking exposure were classified variables, we used Student's t-test and X2 test to verify the difference between cases and controls, respectively. Hardy-Weinberg's equilibrium (HWE) for each SNP in control group was calculated by the goodness-of-fit chi-squared test. Unconditional logistic regression analyses calculated the odds ratios (ORs) and their 95% confidence intervals (CIs) to evaluate the associations between SNP of rs2608053, rs1561927, rs13254990 and the risk of lung cancer and NSCLC. Additionally, we redefined subjects with both the protective genotype and nonsmoking exposure as control group by cross-over study, analyzing the gene-environment interaction to induce lung cancer through additive model and multiplicative model. The sample capacity was estimated by using ''Quanto'' software version 1.2.4 (University of Southern California, Los Angeles, CA). All statistical results were calculated by SPSS 21.0 software (IBM SPSS, Inc. Chicago, IL, USA) and a two-side P value less than 0.05 was considered as significant.

## Results

### Demographic Characteristics

Our case-control study contained 515 cases of lung cancer and 582 controls from health population, which covers non-small cell lung cancer (NSCLC), 278 adenocarcinoma (AD), 176 squamous cell carcinoma (SQ) and 61 small cell lung cancer (SCLC), respectively. Baseline characteristics of both case and control subjects are summarized in table 1. It clearly presented that there were no statistically significant differences in age and gender. The age of cases ranged from 25 to 85 years(mean± SD, 59.63±10.910); the age of controls ranged from 17 to 86 years (mean± SD,58.25±14.612). However, the smoking exposure was 48.0% in the patients, 23.7% in the controls, suggesting that the smoking exposure was a risk factor of lung cancer (P<0.001).Among control group, the genotype frequency distributions of three SNPs were in accordance with Hardy-Weinberg equilibrium (X^2^=0.131, P=0.72 for rs2608053; X^2^=0.559, P=0.455 for rs1561927; X^2^=0.003,P=0.955 for rs13254990) which indicated that the control we selected can represent the general population properly.

### Genotype distribution of PVT1 and lung cancer susceptibility

The unconditional logistic regression analysis was performed to reveal the association with three lncRNAs polymorphisms and the risk of lung cancer as well as NSCLC in Table 2. All the statistical analysis was adjusted by age, gender and smoking status. There was no significantly statistical significance in all genetic (AG vs GG: OR=1.120, 95%CI=0.862-1.455, P=0.396; AA vs GG: OR=1.218, 95%CI=0.711-2.088, P=0.472; Dominant model: OR=1.132, 95%CI=0.880-1.457, P=0.333; Recessive model: OR=1.161, 95%CI=0.686-1.967, P=0.578, adjusted by age, gender and smoking status) models for rs2608053 polymorphism. Similarly, rs1561927 and rs13254990 genetic variation was not significantly statistical significance in all models. Moreover, we did not find the statistically significant relationship between three SNPs and the risk of NSCLC (Table 2). According to pathological types of lung cancer, subgroup analysis was performed by stratification of NCLC into lung adenocarcinoma (AD) and lung squamous cell carcinoma (SQ). To be precise, rs13254990 polymorphisms showed observably results in lung adenocarcinoma. Composed with CC genotypes, the risk of lung adenocarcinoma was significantly increased in TT genotype carriers (adjusted OR=2.095, 95%CI=1.084-4.047, P=0.028). In the recessive model, it also showed a statistically significant difference (TT vs CT+CC: adjusted OR=2.251, 95%CI=1.174-4.318, P=0.015).

In further stratified analysis, the result adjusted by age, gender and smoking exposure was presented in Tables 4, 5 and 6. In age subgroup, neither ≤59 years group nor >59 years group had statistical correlations. Nevertheless, it was noteworthy that we discovered a borderline significance in both AG genotype compared with AA genotype and dominant model of the rs1561927 subgroups of age ≤59 years (P=0.050). Compared with the homozygous CC, the patients with the heterozygous CT had a lower risk of lung cancer in the non-smoking group of rs13254990 (adjusted OR=0.673, 95%CI=0.472-0.959, P=0.028). The same result consisted in NSCLC that individuals carrying genotype CT has the lower NSCLC risk by 0.685-fold compared with the individuals carrying genotype CC (adjusted OR =0.685, 95%CI=0.477-0.984, P=0.040). Besides, there was no significant effect on the risk of lung cancer considering all of the gender subgroup.

### Interaction results between SNPs and smoking status

To investigate the additive interaction of three SNPs and smoking status on lung cancer susceptibility, we implemented a crossover analysis and showed the measure results in Table 7 and 8. For rs1561927, those carriers of AG and GG genotype with smoking exposure had a higher lung cancer risk by 7.108-fold and a higher NSCLC risk by 6.856-foldcompared with the carriers of AA with no smoking exposure (adjusted OR=7.108, 95%CI=2.363-21.376, P=0.000; adjusted OR=6.856, 95%CI=2.237-21.017, P=0.001). For rs2608053, the participants with both AG/AA genotype and smoking were possible to induce lung cancer and NSCLC compared with GG genotype with non-smoking exposure (adjusted OR=5.849, 95%CI=3.691-9.269, P=0.000; adjusted OR=5.472, 95%CI=3.418-8.762, P=0.000). Similar results were also showed in rs13254990 polymorphism. However, three measuring results of rs2608053, rs1561927 and rs13254990 were not statistically significant, which indicated that there was no additive interaction between gene-smoking factor of rs2608053, rs1561927 and rs13254990. In multiplicative interaction analysis, the result showed that there were no statistically meaningful gene-smoking interactions (data were not shown).

In order to further investigate the association of PVT1 with a variety of cancer development, we summarized the effects of PVT1 inhibiting on malignant phenotypes (including proliferation, metastasis, invasion, and apoptosis) and its diverse molecular mechanisms (see Additional file 1: Table S1)^21^, ^28^-^70^. Additionally, PVT1 possesses multifarious molecular functions, including transcriptional dysregulation, pre-mRNA alternative splicing, ceRNA role, epigenetic alterations and transition of cell phenotype through different signaling pathways covering EMT and other pathways. To generalize, PVT1 might exert its molecular function via regulating the expression of related genes and miRNA, affecting carcinogenic signaling pathways, which might induce malignant carcinoma. Moreover, up-regulation of PVT1, comparing with down-regulation of PVT1, might have poor prognosis in various cancers.

## Discussion

This is the first study on the association between lncRNA PVT1 polymorphisms and the susceptibility of lung cancer. It is also the first report on gene-environment interaction between three polymorphisms and smoking status through additive and multiplicative model. The homozygous variant genotype and recessive model of rs13254990 were considered to be related to prominent increase in the risk of lung adenocarcinoma. Additionally, the heterozygote CT genotype of rs13254990 decreased the susceptibility to lung cancer and NSCLC in nonsmokers. Analyzing the measures of additive and multiplicative model, we did not discover the gene-environment interaction.

LncRNAs were detectable and stabilized in body fluids, with a variety of biologic functions, especially ectopic expression, serving as a regulator of tumorigenesis^71^. To be precise, it may affects the proliferation, invasion, migration, metastasis and apoptosis of tumor cells through regulating part of oncogenes and tumor suppressors both at transcriptional and posttranscriptional levels^72^, ^73^. Moreover, it may also induce epithelial-to-mesenchymal transition (EMT) by the PI3K-AKT and miR-143/HMGB1 pathway^74^, ^75^ to promote cancer metastasis. Since lncRNA had played an important part in the emergence, development and even therapy and prognosis of multiple cancers, PVT1, as an emerging gene, got more attention. Up to present, PVT1 was reported to play important roles in the development of lung cancer and NSCLC, by some researchers. Li et al. suggested that PVT1-5 expression was significantly increased to lung cancer tissues and cell lines and was stimulated lung cancer progression by PVT1-5/miR-126/SLC7A5 regulatory network. PVT1-5 positively regulates posttranscriptional expression of SLC7A5 by sponging miR-126, resulting in the cell proliferation^70^. Another study revealed that Yin Yang-1(YY1), a multifunctional transcription factor, bound to the promoter region of PVT1 and motivated its transcription through the consensus YY1 motif to promote cell proliferation, migration and invasion in lung cancer^69^. Likewise, the expression of PVT1 was also increased to NSCLC cell lines, thus promoting the invasive ability of NSCLC cells. As a competitive endogenous RNA(ceRNA), PVT1 suppressed MMP9 expression via competitively binding miR-200a and miR-200b to destroy the reconstruction ability of respiratory tract and lung tissues to induce NSCLC, suggesting that the lncRNA-PVT1-MMP9 axis may be a potential target for NCSLC metastasis^76^. Additionally, Wan et al. illustrated that PVT1 could directly bind to EZH2, a core subunit of the PRC2 complex, to repress the large tumor suppressor kinase 2(LAYS2) transcription in A459 and PC-9 cells. Thus, it induced cell proliferation, induces apoptosis, and promotes cell-cycle arrest in NSCLC, especially lung adenocarcinoma^77^. Consequently, we conducted our case-control study and stratified lung cancer into NSCLC, lung adenocarcinoma and lung squamous cell carcinoma to further analysis the association between PVT1 polymorphisms and lung cancer.

Emerging evidence of Genome-wide association study had demonstrated that SNPs located in PVT1 might be used as susceptibility factors to several cancers. Zhang et al. showed that PVT1 affected cell proliferation in breast cancer via increasing the GG genotype of rs13281615^78^. Furthermore, PVT1 polymorphisms were regulated the prognosis of cancer, too. Zhang et al. illustrated that the presence of rs13281615 G > A polymorphism on PVT1 affected a favorable prognosis in colon cancer patients through modulating the activity of the PVT1/miR-146a/COX2 signaling pathway^79^. For SNPs that conducted in our study, Victor et al. conducted a genome-wide association study (GWAS) of 589 classical Hodgkin Lyphoma (cHL) patients and 5,199 free-controls and replication studies to identify predisposition loci of cHL^25^, suggesting that rs2608053 might be associated with cHL (OR=1.20, 95%CI=1.12-1.28, P=1.16x10^-7^). For rs1561927, it was identified as a risk locus for pancreatic cancer, which located at 455kb telomeric of PVT1, a nongenic region between PVT1 and LINC00977^26^. Moschovis et al. performed a case-control study and indicated that PVT1 rs1561927 G allele was significantly overrepresented in both pancreatic ductal adenocarcinoma (PDAC) cases and pancreatic neuroendocrine tumor (PNET) cases^80^. Additionally, Christine et al. revealed Follicular Lymphoma (FL) susceptibility loci rs13254990 by proceeding a large-scale two-stage GWAS in 4,523 patients and 13,344 controls of European ancestry^27^. Nevertheless, there were some limitations about clarification of causal SNPs and the deep mining of GWAS data. According to it was the first study and the limitations, we chose experimental design and implementation to further explore the relationship between lung cancer and PVT1 polymorphisms. Based on the study of molecular mechanism and PVT1 polymorphisms, we performed the case-control study. In accordance with our study, we found that, as susceptibility loci, the polymorphisms of were associated with the risk of lung cancer in several stratified analysis. It was demonstrated in the analysis that rs13254990 polymorphism increased the risk of lung adenocarcinoma, but it decreased the risk of lung cancer and NCSLC susceptibility in nonsmokers. This is partly due to insufficient capacity and different smoking status. However, rs2608053 and rs1561927 polymorphisms were not related to the risk of lung cancer, which may due to different ethnicities and the lack of data about validate functional experiments.

Several deficiencies in the present case-control study should be emphatically taken into account. First, all study subjects were enrolled from the three hospitals in northeast China with limited sample size, which could lead to Berkson's bias. Secondly, when collecting demographic data, smoking status offered by individuals may result in recall bias and other environmental risk factors of lung cancer could not be found, so these might confuse the associations between SNP and lung cancer susceptibility. Thirdly, functional verification of three SNPs in PVT1 did not perform in present study. Therefore, the underlying mechanisms need to be elucidated with more large-scale subjects across different ethnicities in the future.

## Conclusion

The polymorphism rs13254990 in PVT1 gene is associated with the risk of lung adenocarcinoma in a Chinese northeast population. The interactions between three polymorphisms and smoking status were not statistically significant.

## Supplementary Material

Supplementary figures and tables.

## Figures and Tables

**Table 1:** Demographic Characteristics of patients with lung cancer and controls.

Variables	Cases(%)(N=515)	Controls(%)(N=582)	P-value
Age ,year (mean ± SD)	59.63±10.910	58.25±14.612	0.075
≤ 59	244(47.4)	255(43.8)	0.237
> 59	271(52.6)	327(56.2)	
Gender			
Female	244(47.4)	282(48.5)	0.722
Male	271(52.6)	300(51.5)	
Smoking status			
Ever	247(48.0)	138(23.7)	0.000
Never	268(52.0)	444(76.3)	
Clinical stage			
I,II	128(24.9)		
III	185(35.9)		
IV	87(16.9)		
Other	115(22.3)		
Pathological type			
AD	278(54.0)		
SQ	176(34.2)		
SCLC	61(11.8)		

Abbreviations: AD: lung adenocarcinoma; SQ: lung squamous cell carcinoma; SCLC: small-cell lung cancer.

**Table 2:** Association between the three SNPs and risk of lung cancer and non-small cell lung cancer.

Genotype	Controls (%)(582)	Lung cancer			Non-small cell lung cancer		
		Case (%)(515)	OR^a^ (95%CI)	P^b^-value	Cases (%)(454)	OR^a^ (95%CI)	P^b^-value
Rs2608053							
GG (ref)	328(56.4)	283(55.0)	1.00 (ref)		242(53.3)	1.00 (ref)	
AG	220(37.8)	202(39.2)	1.120(0.862-1.455)	0.396	185(40.7)	1.181(0.903-1.545)	0.224
AA	34(5.8)	30(5.8)	1.218(0.711-2.088)	0.472	27(5.9)	1.256(0,723-2.183)	0.419
AG+AA vs GG			1.132(0.880-1.457)	0.333		1.191(0.919-1.542)	0.186
AA vs AG+GG			1.161(0.686-1.967)	0.578		1.169(0.681-2.006)	0.571
Rs1561927							
AA (ref)	547(94.0)	479(93.0)	1.00 (ref)		421(92.7)	1.00 (ref)	
AG	35(6.0)	35(6.8)	1.302(0.786-2.159)	0.306	33(7.3)	1.347(0.808-2.247)	0.253
GG	0(0.0)	1(0.2)	-	-	0(0.0)	-	-
AG+GG vs AA			1.329(0.804-2.196)	0.267		1.347(0.808-2.247)	0.253
GG vs AG+AA			-	-		-	-
Rs13254990							
CC (ref)	383(65.8)	343(66.6)	1.00 (ref)		303(66.7)	1.00 (ref)	
CT	178(30.6)	144(28.0)	0.842(0.638-1.111)	0.225	126(27.8)	0.848(0.637-1.128)	0.257
TT	21(3.6)	28(5.4)	1.451(0.776-2.714)	0.243	25(5.5)	1.496(0.791-2.830)	0.215
CT+TT vs CC			0.903(0.693-1.176)	0.449		0.913(0.695-1.198)	0.510
TT vs CT+CC			1.529(0.823-2.842)	0.179		1.574(0.837-2.958)	0.159

Abbreviations: SNP: single nucleotide polymorphism; OR: odds ratio; CI: confident interval. Notes: OR^a^ was adjusted by age, gender and smoking status; P^b^-value was adjusted by age, gender and smoking status.

**Table 3:** Association between the three SNPs and risk of lung adenocarcinoma and squamous cell lung cancer.

Genotype	Controls (%)(582)	Lung adenocarcinoma			Lung squamous cell carcinoma		
		Case (%)(278)	OR^a^ (95%CI)	P^b^-value	Cases (%)(176)	OR^a^ (95%CI)	P^b^-value
Rs2608053							
GG (ref)	328(56.4)	147(52.9)	1.00 (ref)		95(54.0)	1.00 (ref)	
AG	220(37.8)	111(39.9)	1.131(0.832-1.538)	0.432	74(42.0)	1.270(0.870-1.856)	0.216
AA	34(5.8)	20(7.2)	1.382(0.758-2.517)	0.291	7(4.0)	0.996(0.405-2.446)	0.993
AG+AA vs GG			1.163(0.867-1.562)	0.313		1.239(0.857-1.790)	0.254
AA vs AG+GG			1.311(0.730-2.353)	0.364		0.900(0.372-2.176)	0.815
Rs1561927							
AA (ref)	547(94.0)	252(90.6)			169(96.0)		
AG	35(6.0)	26(9.4)	1.637(0.950-2.820)	0.076	7(4.0)	0.759(0.317-1.813)	0.534
GG	0(0.0)	0(0.0)	-	-	0(0.0)	-	-
AG+GG vs AA			1.637(0.950-2.820)	0.076		0.759(0.317-1.813)	0.534
GG vs AG+AA			-	-		-	-
Rs13254990							
CC (ref)	383(65.8)	187(67.3)	1.00 (ref)		116(65.9)	1.00 (ref)	
CT	178(30.6)	70(25.2)	0.782(0.560-1.093)	0.150	56(31.8)	1.030(0.694-1.529)	0.883
TT	21(3.6)	21(7.6)	2.095(1.084-4.047)	0.028*	4(2.3)	0.570(0.180-1.806)	0.339
CT+TT vs CC			0.913(0.669-1.246)	0.565		0.978(0.666-1.436)	0.911
TT vs CT+CC			2.251(1.174-4.318)	0.015*		0.564(0.179-1.777)	0.328

Abbreviations: SNP: single nucleotide polymorphism; OR: odds ratio; CI: confident interval; * indicates statistical significance (*P*<0.05). Notes: OR^a^ was adjusted by age, gender and smoking status; P^b^-value was adjusted by age, gender and smoking status.

**Table 4:** Stratified analyses of association between the three SNPs and lung cancer risk by average age.

Genotype	Age	Controls (%)(582)	Lung cancer			Non-small cell lung cancer		
			Case(%)(515)	OR^a^(95%CI)	P^b^-value	Cases(%)(454)	OR^a^(95%CI)	P^b^-value
Rs2608053								
GG	≤59 years	143(56.1)	139(57.0)	1.00 (ref)		108(54.3)	1.00 (ref)	
AG		97(38.0)	92(37.7)	0.832(0.555-1.249)	0.375	80(40.2)	0.889(0.581-1.361)	0.589
AA		15(5.9)	13(5.3)	0.938(0.403-2.186)	0.882	11(5.5)	0.964(0.398-2.333)	0.935
AG+AA vs GG				0.845(0.572-1.248)	0.398		0.899(0.597-1.353)	0.608
AA vs AG+GG				1.011(0.442-2.316)	0.979		1.013(0.426-2.405)	0.977
GG	>59 years	185(56.6)	144(53.1)	1.00 (ref)		134(52.5)	1.00 (ref)	
AG		123(37.6)	110(40.6)	1.354(0.943-1.945)	0.101	105(41.2)	1.388(0.962-2.002)	0.080
AA		19(5.8)	17(6.3)	1.545(0.748-3.194)	0.240	16(6.3)	1.580(0.758-3.296)	0.222
AG+AA vs GG				1.378(0.973-1.953)	0.071		1.412(0.992-2.009)	0.055
AA vs AG+GG				1.355(0.668-2.747)	0.400		1.369(0.669-2.799)	0.389
Rs1561927								
AA	≤59 years	244(95.7)	227(93.0)	1.00 (ref)		183(92.0)	1.00 (ref)	
AG		11(4.3)	16(6.6)	1.920(0.814-4.530)	0.136	16(8.0)	2.369(1.002-5.604)	0.050*
GG		0(0.0)	1(0.4)	-	-	0(0.0)	-	-
AG+GG vs AA				1.998(0.855-4.669)	0.110		2.369(1.002-5.604)	0.050*
GG vs AG+AA				-	-		-	-
AA	>59 years	303(92.7)	255(93.0)	1.00 (ref)		238(93.3)	1.00 (ref)	
AG		24(7.3)	19(7.0)	1.252(0.649-2.417)	0.503	17(6.7)	1.144(0.581-2.251)	0.697
GG		0(0.0)	0(0.0)	-	-	0(0.0)	-	-
AG+GG vs AA				1.252(0.649-2.417)	0.503		1.144(0.581-2.251)	0.697
GG vs AG+AA				-	-		-	-
Rs13254990								
CC	≤59 years	166(65.1)	166(68.0)	1.00 (ref)		134(67.3)	1.00(ref)	
CT		80(31.4)	64(26.2)	0.753(0.490-1.159)	0.197	54(27.1)	0.790(0.503-1.241)	0.306
TT		9(3.5)	14(5.7)	1.699(0.646-4.467)	0.282	11(5.5)	1.795(0.654-4.927)	0.256
CT+TT vs CC				0.840(0.558-1.263)	0.402		0.879(0.572-1.350)	0.555
TT vs CT+CC				1.849(0.710-4.815)	0.208		1.928(0.709-5.240)	0.198
CC	>59 years	217(66.4)	177(65.3)	1.00 (ref)		169(66.3)	1.00(ref)	
CT		98(30.0)	80(29.5)	0.958(0.657-1.398)	0.826	72(28.2)	0.913(0.621-1.341)	0.641
TT		12(3.7)	14(5.2)	1.281(0.546-3.003)	0.569	14(5.5)	1.335(0.572-3.117)	0.504
CT+TT vs CC				0.994(0.693-1.426)	0.975		0.960(0.666-1.384)	0.826
TT vs CT+CC				1.298(0.558-3.018)	0.545		1.373(0.593-3.179)	0.460

Abbreviations: SNP: single nucleotide polymorphism; OR: odds ratio; CI: confident interval; * indicates statistical significance (*P*<0.05). Notes: OR^a^ was adjusted by age, gender and smoking status; P^b^-value was adjusted by age, gender and smoking status.

**Table 5:** Association of the three SNPs with lung cancer risks and non-small-cell lung cancer risks in female and male populations.

Genotype	Gender	Controls (%)(582)	Lung cancer			Non-small cell lung cancer		
			Case(%)(515)	OR^a^(95%CI)	P^b^-value	Cases(%)(454)	OR^a^(95%CI)	P^b^-value
Rs2608053								
GG	Female	151(53.5)	121(49.6)	1.00 (ref)		108(48.2)	1.00 (ref)	
AG		111(39.4)	107(43.9)	1.249(0.862-1.809)	0.240	101(45.1)	1.339(0.916-1.958)	0.131
AA		20(7.1)	16(6.6)	1.169(0.577-2.368)	0.664	15(6.7)	1.246(0.607-2.559)	0.549
AG+AA vs GG				1.237(0.867-1.765)	0.241		1.325(0.920-1.909)	0.130
AA vs AG+GG				1.058(0.533-2.098)	0.873		1.089(0.542-2.187)	0.811
GG	Male	177(59.0)	162(59.8)	1.00 (ref)		134(58.3)	1.00 (ref)	
AG		109(36.3)	95(35.1)	0.986(0.677-1.436)	0.941	84(36.5)	1.034(0.701-1.524)	0.868
AA		14(4.7)	14(5.2)	1.304(0.562-3.026)	0.536	12(5.2)	1.312(0.549-3.132)	0.541
AG+AA vs GG				1.019(0.709-1.463)	0.920		1.062(0.730-1.544)	0.752
AA vs AG+GG				1.311(0.572-3.005)	0.522		1.295(0.550-3.049)	0.555
Rs1561927								
AA	Female	261(92.6)	223(91.4)	1.00 (ref)		205(91.5)	1.00 (ref)	
AG		21(7.4)	21(8.6)	1.334(0.702-2.536)	0.379	19(8.5)	1.294(0.670-2.500)	0.443
GG		0(0.0)	0(0.0)	-	-	0(0.0)	-	-
AG+GG vs AA				1.334(0.702-2.536)	0.379		1.294(0.670-2.500)	0.443
GG vs AG+AA				-	-		-	-
AA	Male	286(95.3)	256(94.5)	1.00 (ref)		216(93.9)	1.00 (ref)	
AG		14(4.7)	14(5.2)	1.381(0.608-3.319)	0.441	14(6.1)	1.585(0.700-3.588)	0.269
GG		0(0.0)	1(0.4)	-	-	0(0.0)	-	-
AG+GG vs AA				1.460(0.651-3.274)	0.358		1.585(0.700-3.588)	0.269
GG vs AG+AA				-	-		-	-
Rs13254990								
CC	Female	183(64.9)	168(68.9)	1.00 (ref)		155(69.2)	1.00(ref)	
CT		91(32.3)	64(26.2)	0.687(0.460-1.025)	0.066	59(26.3)	0.691(0.458-1.042)	0.078
TT		8(2.8)	12(4.9)	1.223(0.455-3.289)	0.689	10(4.5)	1.176(0.423-3.265)	0.756
CT+TT vs CC				0.730(0.497-1.073)	0.109		0.731(0.493-1.083)	0.118
TT vs CT+CC				1.369(0.513-3.650)	0.530		1.313(0.476-3.617)	0.599
CC	Male	200(66.7)	175(64.6)	1.00 (ref)		148(64.3)	1.00(ref)	
CT		87(29.0)	80(29.5)	1.011(0.681-1.499)	0.958	67(29.1)	1.025(0.680-1.546)	0.905
TT		13(4.3)	16(5.9)	1.541(0.673-3.527)	0.306	15(6.5)	1.708(0.741-3.940)	0.209
CT+TT vs CC				1.075(0.740-1.561)	0.706		1.109(0.752-1.634)	0.602
TT vs CT+CC				1.536(0.677-3.485)	0.304		1.695(0.742-3.872)	0.211

Abbreviations: SNP: single nucleotide polymorphism; OR: odds ratio; CI: confident interval; * indicates statistical significance (*P*<0.05). Notes: OR^a^ was adjusted by age, gender and smoking status; P^b^-value was adjusted by age, gender and smoking status.

**Table 6:** Stratified analyses of the three SNPs with lung cancer risks and non-small-cell lung cancer risks by smoking status.

Genotype	Smoking exposure	Controls (%)(582)	Lung cancer			Non-small cell lung cancer		
			Case(%)(515)	OR^a^(95%CI)	P^b^-value	Cases(%)(454)	OR^a^(95%CI)	P^b^-value
Rs2608053								
GG	Ever	83(60.1)	152(61.5)	1.00 (ref)		123(59.7)	1.00 (ref)	
AG		52(37.7)	84(34.0)	0.894(0.569-1.405)	0.628	74(35.9)	1.026(0.639-1.646)	0.917
AA		3(2.2)	11(4.5)	2.177(0.586-8.083)	0.245	9(4.4)	2.243(0.581-8.660)	0.241
AG+AA vs GG				0.966(0.623-1.500)	0.879		1.096(0.692-1.737)	0.696
AA vs AG+GG				2.267(0.617-8.332)	0.218		2.222(0.582-8.477)	0.243
GG	Never	245(55.2)	131(48.9)	1.00 (ref)			1.00 (ref)	
AG		168(37.8)	118(44.0)	1.279(0.927-1.764)	0.135	119(48.0)	1.314(0.946-1.827)	0.104
AA		31(7.0)	19(7.1)	1.109(0.598-2.059)	0.742	111(44.7)	1.153(0.615-2.162)	0.657
AG+AA vs GG				1.252(0.920-1.705)	0.153	18(7.3)	1.289(0.940-1.768)	0.115
AA vs AG+GG				0.995(0.546-1.815)	0.987		1.020(0.554-1.879)	0.949
Rs1561927								
AA	Ever	133(96.4)	236(95.5)	1.00 (ref)		196(95.1)	1.00 (ref)	
AG		5(3.6)	10(4.0)	1.255(0.406-3.876)	0.693	10(4.9)	1.609(0.514-5.041)	0.414
GG		0(0.0)	1(0.4)	-	-	0(0.0)	-	-
AG+GG vs AA				1.399(0.462-4.238)	0.552		1.609(0.514-5.041)	0.414
GG vs AG+AA				-	-		-	-
AA	Never	414(93.2)	243(90.7)	1.00 (ref)		225(90.7)	1.00 (ref)	
AG		30(6.8)	25(9.3)	1.383(0.787-2.429)	0.260	23(9.3)	1.367(0.769-2.430)	0.287
GG		0(0.0)	0(0.0)	-	-	0(0.0)	-	-
AG+GG vs AA				1.383(0.787-2.429)	0.260		1.367(0.769-2.430)	0.287
GG vs AG+AA				-	-		-	-
Rs13254990								
CC	Ever	93(67.4)	151(61.1)	1.00 (ref)		126(61.2)	1.00(ref)	
CT		39(28.3)	81(32.8)	1.252(0.777-2.016)	0.355	67(32.5)	1.273(0.772-2.099)	0.345
TT		6(4.3)	15(6.1)	1.304(0.471-3.611)	0.609	13(6.3)	1.433(0.502-4.086)	0.501
CT+TT vs CC				1.259(0.800-1.981)	0.319		1.295(0.805-2.082)	0.286
TT vs CT+CC				1.214(0.443-3.323)	0.707		1.327(0.471-3.740)	0.592
CC	Never	290(65.3)	192(71.6)	1.00 (ref)		177(71.4)	1.00(ref)	
CT		139(31.3)	63(23.5)	0.673(0.472-0.959)	0.028*	59(23.8)	0.685(0.477-0.984)	0.040*
TT		15(3.4)	13(4.9)	1.465(0.670-3.206)	0.339	12(4.8)	1.471(0.662-3.268)	0.344
CT+TT vs CC				0.744(0.532-1.039)	0.083		0.755(0.536-1.063)	0.107
TT vs CT+CC				1.639(0.754-3.563)	0.213		1.638(0.742-3.615)	0.222

Abbreviations: SNP: single nucleotide polymorphism; OR: odds ratio; CI: confident interval; * indicates statistical significance (*P*<0.05). Notes: OR^a^ was adjusted by age and gender; P^b^-value was adjusted by age, gender and smoking status.

**Table 7:** Crossover analysis of interaction between rs2608053, rs1561927 and rs13254990 risk genotypes and smoking exposure.

SNPs	Genotype	Smoking exposure	Controls (%)(582)	Lung cancer			Non-small cell lung cancer		
				Cases(%)(515)	OR^a^(95%CI)	P^b^-value	Cases(%)(454)	OR^a^(95%CI)	P^b^-value
Rs2608053	GG	Never	245(42.1)	131(25.4)	1.00(ref)		119(26.2)	1.00(ref)	
	AG+AA	Never	199(34.2)	137(26.6)	1.242(0.911-1.694)	0.170	129(28.4)	1.275(0.928-1.753)	0.134
	GG	Ever	83(14.3)	152(29.5)	6.175(4.073-9.364)	0.000*	123(27.1)	5.256(3.429-8.054)	0.000*
	AG+AA	Ever	55(9.5)	95(18.4)	5.849(3.691-9.269)	0.000*	83(18.3)	5.472(3.418-8.762)	0.000*
Rs1561927	AA	Never	414(71.1)	243(47.2)	1.00(ref)		225(49.6)	1.00(ref)	
	AG+GG	Never	30(5.2)	25(4.9)	1.342(0.762-2.363)	0.309	23(5.1)	1.326(0.743-2.367)	0.339
	AA	Ever	133(22.9)	236(45.8)	5.537(3.878-7.904)	0.000*	196(43.2)	4.806(3.343-6.910)	0.000*
	AG+GG	Ever	5(0.9)	11(2.1)	7.108(2.363-21.376)	0.000*	10(2.2)	6.856(2.237-21.017)	0.001*
Rs13254990	CC+CT	Never	429(73.7)	255(49.5)	1.00(ref)		236(52.0)	1.00(ref)	
	TT	Never	15(2.6)	13(2.5)	1.689(0.773-3.692)	0.189	12(2.6)	1.697(0.763-3.776)	0.195
	CC+CT	Ever	132(22.7)	232(45.0)	5.555(3.880-7.953)	0.000*	193(42.5)	4.833(3.354-6.966)	0.000*
	TT	Ever	6(1.0)	15(2.9)	7.238(2.680-19.549)	0.000*	13(2.9)	6.740(2.439-18.626)	0.000*

Abbreviations: SNP: single nucleotide polymorphism; OR: odds ratio; CI: confident interval; * indicates statistical significance (*P*<0.05). Notes: OR^a^ was adjusted by age and gender; P^b^-value was adjusted by age and gender.

**Table 8:** Addictive interaction between rs2608053, rs1561927, rs13254990 risk genotypes and smoking exposure.

SNPs	Measure	Lung cancer		Non-small cell lung cancer	
		Estimate	95%CI	Estimate	95%CI
Rs2608053	RERI	-0.568	-3.174 to 2.038	-0.059	-2.481 to 2.364
	AP	-0.097	-0.565 to 0.371	-0.011	-0.456 to 0.435
	S	0.895	0.537 to 1.492	0.987	0.576 to 1.691
Rs1561927	RERI	-1.272	-2.441 to -0.103	-1.243	-2.415 to -0.072
	AP	-0.948	-2.239 to 0.343	-0.937	-2.247 to 0.373
	S	0.212	0.021 to 2.106	0.208	0.018 to 2.357
Rs13254990	RERI	-0.963	-2.502 to 0.577	-0.910	-2.475 to 0.656
	AP	-0.570	-1.871 to 0.731	-0.536	-1.835 to 0.763
	S	0.417	0.059 to 2.944	0.434	0.060 to 3.153

Abbreviations: CI: confidence interval; RERI: relative excess risk due to interaction; AP: attributable proportion due to interaction; S: synergy index.

## Abbreviations

LncRNA: long non-coding RNA
PVT1: plasmacytoma variant translocation 1
SNP: single nucleotide polymorphism
NSCLC: non-small cell lung cancer
AD: lung adenocarcinoma
SQ: lung squamous cell carcinoma
SCLC: small cell lung cancer
HWE: Hardy-Weinberg equilibrium
95% CI: 95% confidence interval
OR: odds ratio
Real-Time PCR: real-time polymerase chain reaction
GWAS: genome-wide association study
