# Supplementary material for: Polymorphisms in the PVT1 Gene and Susceptibility to the Lung Cancer in a Chinese Northeast Population: a Case-control Study
Source: J Cancer. 2020 Jan 1;11(2):468–78. doi: 10.7150/jca.34320 (PMC6930418; doi:10.7150/jca.34320)
Supplement: Supplementary file 1 — Supplementary figures and tables. [file jcav11p0468s1.pdf]

Additional file 1

Table S1 Research advances of PVT1 roles in human cancers (↑indicates increase; ↓indicates decrease)

Cancer type	Expression effect	Clinical features of up-regulated PVT1	Transition of cell phenotype of PVT1 inhibition	Carcinogenic mechanisms	References
Renal cell carcinoma	Up-regulated	-	↓invasion/migration;↑apoptosis	Inhibition of miR-16-5p partly abrogates apoptosis and inhibition of EMT caused by silencing of PVT1; PVT1 acts as the sponge of miR-16-5p	**[28]**
Clear cell renal cell carcinoma	Up-regulated	↓OS/DFS	↑ G0/G1 phase;↓S phase; ↓ migration/invasion; ↓ proliferation;↑apoptosis/cell cycle arrest	PVT1 up-regulates BMI1, ZEB1 and ZEB2 levels by sponging miR-200s; PVT1 regulates the activation of the EGFR pathway	**[29, 30]**
Prostate cancer	Up-regulated	↓OS/DFS	↓ proliferation migration/tumor growth/ metastasis;↑apoptosis	The expression of cleaved caspase-3 and caspase-9 up-regulated and down-regulation of c-Myc by knockdown PVT1; Knockdown of PVT1 decreased the phosphorylation of p38 in DU145 cells to inhibits proliferation and migration in prostate cancer; PVT1 as a ceRNA to reduce the expression of miR-186 in PCa cell lines and promote Twist1 expression via suppressing miR-186; PVT1 induces EMT by inducing up-regulation of Twist1 expression; PVT1 regulated miR - 146a expression through inducing the methylation of CpG Island	**[21, 31-33]**
Gastric cancer	Up-regulated	↓ DFS/DSS/OS; ↑ TNM stage;	↓ metastasis/invasion/proliferation; ↑ apoptosis	PVT1 promoter binds to FOXM1; PVT1 act as a FOXM1-mediated manner to facilitate tumor proliferation and metastasis; PVT1 regulate the expression of p15 and p16 by targeting E2H2; PVT1 activates the STAT3 signaling pathway and elevates VEGFA expression to stimulate angiogenesis; PVT1 down-regulates the miR-186 expression and inhibits the HIF-1α expression in GC cells; PVT1 increases the expression of CD151 and FGF2 through regulating miR-152	**[34-39]**
Glioma	Up-regulated	↓ OS	↓migration/invasion/proliferation/ G0/G1 phase;↑apoptosis	Over-expression of PVT1 inhibits the expression of miR-200a; PVT1 acts as a sponge of miR-128-3p and regulates the BMP signaling pathway downstream proteins BMP2 and BMP4 through regulating GREM1; PVT1 induces proliferation and invasion of glioma by up-regulating EZH2 protein expression; Over-expression of PVT1 increases the expression of Atg7 and Beclin1 by targeting miR-186	**[40-44]**
Colorectal cancer	Up-regulated	↓ DFS/OS	↓migration/invasion/proliferation	PVT1 induce cell migration, invasion, and proliferation via sponging miR-26b; PVT1 serves as a ceRNA and negatively regulates miR-455 expression; The RUNX2/PVT1/miR-455 regulatory axis regulates cell proliferation, migration and invasion; PVT1 induces metastasis and	**[45-48]**

				proliferation via suppressing miR-30d-5p/RUNX2 axis	
Breast cancer	Up-regulated	↓ OS;↑DFS	↓proliferation	SOX2 activates PVT1 expression promoting breast cancer progression through EMT pathway; Down-regulation of PVT1 augments p21 mRNA and protein expression; PVT1 binds with KLF5 and increases its stability via BAP1; MYC and PVT1 synergize to increase level of RSPO1	**[49-52]**
Ovarian cancer	Up-regulated	↓ OS/PFS;	↓proliferation/migration/invasion; ↑G0/G1 phase/S phase/apoptosis	PVT1 inhibits miR-214 expression by interacting with EZH2; Knockdown of PVT1 inhibits cell proliferation, migration and invasion through negative modulation miR-133a; PVT1 promotes tumorigenesis via SOX2	**[53-56]**
Bladder cancer	Up-regulated	-	↓proliferation	Knockdown of PVT1increases the expression of miR-31and decreases the positive rate of CDK1	**[57]**
Osteosarcoma	Up-regulated	↓ OS	↓proliferation/ S phase; ↑G0/G1 phase/apoptosis	PVT1 promotes glycolysis and the expression of HK2; PVT1 regulates miR-497/HK2 axis to promote cell proliferation and invasion; Over-expression of PVT1 decreases the mRNA expression level of miR-195 to promote cell cycle arrest and apoptosis	**[58, 59]**
Pancreatic cancer	Up-regulated	↓ OS	↓proliferation/migration	PVT1 acts as an sponge through competing for miR-448 binding to regulate the miRNA target SERBP1; Over-expression of PVT1 increases Snail and ZEB1 expression while decreases p21 expression; PVT1 induces EMT and cell proliferation and migration through down-regulating p21	**[60, 61]**
Cervical cancer	Up-regulated	↑ tumor size; ↓ OS	↓proliferation/migration/invasion/S phase;↑G0/G1 phase/apoptosis	PVT1 interacts with EZH2 and the complex anchors in the promoter region of miR-195; PVT1 binds with E2Z2 to regulate miR-200b expression	**[62, 63]**
Nasopharyngeal carcinoma	Up-regulated	↓ OS/RFS	↓proliferation;↑apoptosis	Knockdown of PVT1 diminish DNA repair ability through the ATM–p53 pathway	**[64]**
Esophageal squamous cell carcinoma	Up-regulated	-	↓proliferation/migration/tumor growth	PVT1 promotes ESCC progression via functioning as a molecular sponge for miR-203 and LASP1	**[65]**
Hepatocellular carcinoma	Up-regulated	↓ OS	↓proliferation/migration/invasion; ↑apoptosis	PVT1 inhibits effect on YAP1 by acting as an endogenous sponge for miR-186-5p; PVT1 inhibits miR-214 expression by interacting with EZH2; Knockdown of PVT1 inhibits the expression level of EZH2 and MDM2 as well as promotes P53 expression	**[66-68]**
Lung cancer	Up-regulated	-	↓proliferation;↑apoptosis	YY1 bound to the promoter region of PVT1 and motivate its transcription to regulate tumorigenesis; PVT1-5 positively regulates posttranscriptional expression of SLC7A5 by sponging miR-126	**[69, 70]**
